# Comprehensive Genomic Profiling of Circulating Tumor DNA in Patients with Previously Treated Metastatic Colorectal Cancer: Analysis of a Real-World Healthcare Claims Database

**DOI:** 10.3390/curroncol29050277

**Published:** 2022-05-09

**Authors:** Yoshiaki Nakamura, Steven Olsen, Nicole Zhang, Jiemin Liao, Takayuki Yoshino

**Affiliations:** 1 Department of Gastroenterology and Gastrointestinal Oncology, National Cancer Center Hospital East, Kashiwa 277-8577, Japan; yoshinak@east.ncc.go.jp (Y.N.); tyoshino@east.ncc.go.jp (T.Y.); 2 Translational Research Support Section, National Cancer Center Hospital East, Kashiwa 277-8577, Japan; 3 Department of Medical Affairs, Guardant Health Asia, Middle East, Africa, Inc., Tokyo Port City Takeshiba Office Tower 9th Floor, 1-7-1 Kaigan, Minato-ku, Tokyo 105-7590, Japan; 4 Department of Outcomes and Evidence, Guardant Health, Inc., Redwood City, CA 94063, USA; niczhang@guardanthealth.com (N.Z.); jiliao@guardanthealth.com (J.L.)

**Keywords:** actionable genomic alterations, colorectal cancer, ctDNA profiling, real-world, targeted therapy

## Abstract

We used a real-world database (GuardantINFORM^TM^) to analyze the treatment choices for patients with mCRC who underwent next-generation sequencing of circulating tumor DNA (ctDNA) using a commercially available test (Guardant360^®^) after first- or second-line therapy. From 18,875 patients with claims for CRC, 1064 had confirmed metastatic disease and sufficient histories for analysis (median age 59 years, 44.8% female, 44.5% left-sided). ctDNA was detectable for 997/1064 (93.7%) patients. Clinically actionable molecular profiles were present for 507/1064 (47.7%) patients, including those who had not received targeted therapy in the previous line (410/926, 44.3%). Second- or third-line targeted therapies were administered to 338/1064 patients (31.8%) and were considered matched for 193/338 (57.1%) patients. Therapies administered after testing were informed by the ctDNA results in 56.7% of patients overall (603/1064). Time to treatment discontinuation was most favorable for patients with a clinically actionable ctDNA profile who received matched therapy. This analysis demonstrates the real-world clinical value of plasma-based comprehensive genomic profiling for selecting appropriate molecular-targeted therapies in mCRC patients with disease progression after first- or second-line therapy.

## 1. Introduction

Advancements in molecular-targeted therapies and biomarker detection have improved in recent years [1,2]. Such technologies have been successfully applied to people living with metastatic colorectal cancer (mCRC). In particular, the treatment of mCRC has progressed significantly over the past 20 years, with the addition of new chemotherapy to fluoropyrimidine backbones along with the introduction of agents that target vascular endothelial growth factor (VEGF) and molecular aberrations of the cancer cells themselves. Such advancements in molecular-targeted therapies and biomarker detection have resulted in improvements in tumor response rates and overall survival [3,4]. The presence of specific biomarkers in these tumors can predict the response to targeted therapies. For example, tumors with wild-type *KRAS* and *NRAS* (which account for 30%–50% of cases) [5,6,7,8,9,10,11,12] are more likely to respond to EGFR-targeted therapies than tumors with mutations in either of these genes [13,14]. Other examples of targeted therapies for mCRC include immune checkpoint inhibitors (ICIs) for microsatellite-instability-high (MSI-H) tumors [15], tyrosine kinase inhibitors for *BRAF* V600E [16], and ERBB2-targeted therapies for *ERBB2* amplification [17,18]. Therefore, US treatment guidelines recommend testing for mutations in *KRAS*, *NRAS*, and *BRAF*; *ERBB2* amplification; *NTRK1/2/3* rearrangements; and MSI-high status in patients with advanced CRC [19].

Resistance mutations may emerge after treatment with targeted therapies. For example, EGFR-targeted therapy may lead to alterations such as *KRAS*, *NRAS*, *BRAF*, and *EGFR* extracellular domain mutations or *ERBB2* amplification [20,21,22]. Some of these can be successfully treated with additional targeted therapies, such as trastuzumab-based therapies or trastuzumab deruxtecan (DS-8201) for *ERBB2* amplification [18,23,24,25,26,27]. However, for tumors lacking these mechanisms of resistance, repeated treatment with EGFR-targeted therapy is feasible and effective [20].

Therefore, genomic assessment of mCRC after progression on initial treatment can guide therapy selection. While tissue biopsies have traditionally been used for such assessment, access to tumor tissue for biomarker testing may be challenging. Furthermore, archived tissue samples may not represent the current state of the tumor and it may be difficult to obtain a fresh tissue biopsy.

Recent advances in next-generation sequencing (NGS) of circulating tumor DNA (ctDNA) have made liquid biopsy a viable testing modality for sequencing mCRC after disease progression. Comprehensive genomic profiling of ctDNA can detect mutations, gene rearrangements, and amplification in genes that may be targets for subsequent therapy or provide information on resistance mechanisms to help clinicians avoid ineffective therapies [28,29].

In this study, we sought to evaluate how clinicians apply ctDNA testing results when making treatment decisions for patients with mCRC that had progressed on prior therapy. Using a real-world database (GuardantINFORM^TM^, Guardant Health, Redwood City, CA, USA), we analyzed the treatment choices for patients with mCRC who underwent NGS-based ctDNA testing (Guardant360^®^, Guardant Health) after completing first- or second-line therapy. The goal was to evaluate the real-world application of ctDNA testing and its ability to identify clinically relevant genomic alterations and how such findings influence the subsequent treatment decisions and clinical outcomes for people living with mCRC.

## 2. Methods

### 2.1. Ethics

The GuardantINFORM database is fully compliant and deidentified in accordance with the US patient confidentiality requirements set forth in Sections 164.514 (a)–(b)1ii of the Health Insurance Portability and Accountability Act (HIPAA) regarding the determination and documentation of statistically deidentified data. Institutional Review Board approval to conduct this study was not required because this study used deidentified patient records and did not involve the collection, use, or transmission of individually identifiable data.

### 2.2. Data Source and Extraction

We used secondary data from the GuardantINFORM database, which comprises anonymized genomic data for patients with advanced stage solid tumors in the US who underwent testing with a Guardant360 assay. This assay is certified according to Clinical Laboratory Improvement Amendments and accredited by the College of American Pathologists and has been approved by the US Food and Drug Administration (FDA) and New York State Department of Health for clinical testing of patients with advanced stage (stage III–IV) solid tumors. The assay utilizes hybrid capture technology and NGS to identify genomic alterations in 74 genes [30,31]. The panel covers all coding exons in *KRAS*, *NRAS*, and *BRAF*, and detects *ERBB2* amplification, *NTRK1* fusion, and MSI-high status.

In addition to the genetic data, the GuardantINFORM database includes structured commercial payer claims data collected from inpatient and outpatient facilities in academic and community settings. Claims data are provided through a commercial agreement with a data aggregator (the ‘parent dataset’), which collates high-quality industry-standard sources of anonymized patient level data and a further 150 fully privileged payer complete datasets that include a combination of open and closed claims. By percentage, the prescription claim coverage of the full parent dataset by payer type is: Commercial 78%, Medicare 0%, Medicare Advantage 16%, and Medicaid 6%. However, it does not capture clinical features that are not coded as claims, such as the results of tumor biomarker testing from other sources and the clinical response to cancer treatment. Deaths are sourced from third-party providers and aggregated with administrative claims data; for at least half of the CDC-reported deaths in the country, the parent dataset has an encounter within ≤1 month of the date of death. GuardantINFORM is refreshed every quarter. At the time of this study, GuardantINFORM contained records for more than 190,000 patients across 60 cancer types, representing approximately 7000 oncologists from US-based academic and community cancer practices.

### 2.3. Study Population

For this study, we included all CRC patients who underwent testing between 1 June 2014 and 30 September 2021, with a Guardant360 assay performed within 90 days prior to initiating second or third-line systemic therapy, had a 6-month clean window of claims information (≥2 medical or pharmacy claims) without treatment prior to the first observed therapy date, and had claims data indicating the presence of metastatic disease. The clean window was implemented to ensure that the first observed systemic therapy date corresponds to first-line treatment. If patients underwent ctDNA testing more than once after initial treatment, data from the test conducted immediately after the earliest line of targeted therapy were analyzed; otherwise, the earlier test was used regardless of prior therapy.

### 2.4. Lines of Therapy

The patients’ treatment regimens were reconstructed over the study period based on the claims data. All systemic cancer therapies recommended for mCRC in the NCCN guidelines [19] were included. The same regimens used within 90 days from the end of one to the start of the next administration date were combined and considered the same line of therapy. Any treatment modifications or additions to targeted therapies more than 60 days after treatment initiation were considered a new line of therapy, whereas changes to chemotherapies more than 15 days after treatment initiation were considered a new line of therapy. Changes in treatments involving fluorouracil or bevacizumab were not considered as new therapy. Discontinuing a medicine without adding a new medicine to a regimen was not considered a new line of therapy.

### 2.5. Genomic Profiling

Clinically informative genomic alterations were those classified as Therapeutic Levels 1, 2 or R1 by OncoKB [32]; namely, *KRAS* and *NRAS* point mutations, *BRAF* V600E, *ERBB2* amplification, *NTRK1* fusion, and MSI-high status. The Guardant360 assay used at the time of data collection did not include *NTRK2* or *NTRK3* fusions, nor did it assess tumor mutation burden status.

For determination of actionable genomic profiles and molecularly informed therapy, only the results of Guardant360 tests were considered because the GuardantINFORM database did not include biomarker results for other testing modalities. Samples with detectable ctDNA but neither *KRAS* nor *NRAS* mutations were classified as having an actionable molecular profile, and patients with such profiles were categorized as appropriate candidates for EGFR-targeted therapy except in the case of concurrent *BRAF* V600E or primary tumor location in the right colon [33]. Immune checkpoint inhibitors were considered molecularly informed only if MSI-high were detected. BRAF-directed therapies were considered informed for *BRAF* V600E, ERBB2-targeted therapies were considered informed for *ERBB2* amplification, and NTRK inhibitors were considered informed for *NTRK1* fusions. Chemotherapy and/or VEGF-targeted therapy were classified as molecularly informed in the absence of *ERBB2* amplification, *BRAF* V600E, *NTRK1* fusion, and MSI-high or the presence of a mutation in *KRAS* or *NRAS* without other actionable biomarkers. Non-targeted therapy was also considered informed for patients with right-sided tumors and ctDNA profiles lacking *RAS* mutations or other actionable alterations. Throughout this manuscript, “targeted therapy” refers to systemic anti-cancer agents that are targeted primarily against aberrant proteins expressed by cancer cells or to immune checkpoint inhibitors; VEGF-targeted therapy is not included in this definition.

### 2.6. Patient Outcomes

As a surrogate for progression-free survival, we evaluated the time to treatment discontinuation (TTD) for each line of therapy, which was defined as the time from the first day therapy to the estimated last day of therapy, the last claim activity date, or death, whichever occurred first. Patients with a last claim activity date prior to the end of therapy or <90 days after the end of therapy were censored due to lack of follow-up information. We also evaluated overall survival, which was defined as the time from the ctDNA report date prior to the start of second- or third-line therapy (for second- or third-line therapy patients, respectively) until death. Patients were censored at last claim activity date.

### 2.7. Statistical Analysis

All patients in the study population were included in the baseline descriptive statistics. Means, medians, and standard deviations were calculated for continuous variables whereas counts and proportions were calculated for categorical variables. Proportions were compared using the two proportion *z*-test or Fisher’s Exact test for small samples.

The TTD for anti-cancer therapies administered after ctDNA testing was evaluated using the Kaplan–Meier method for the following four subgroups of patients: (1) patients with a clinically actionable genomic profile who received the matched therapy, (2) patients with a clinically actionable genomic profile who did not receive a matched therapy, (3) patients without a clinically actionable genomic profile, and (4) patients in whom ctDNA was not detected. Log-rank tests were used to compare TTD across these subgroups. In addition, a Cox proportional hazards model was performed with these four subgroups to generate pairwise hazard ratios (HRs) with 95% confidence intervals. A *p*-value of <0.05 was considered statistically significant.

Subgroup analyses were performed for patients who received EGFR-targeted therapy or immune checkpoint inhibitors, and the TTD was compared between patients whose ctDNA profiles supported the choice of the targeted treatment (matched) and those whose profiles did not support such treatment (unmatched).

All analyses were conducted using SAS software package 9.4 (SAS Institute, Cary, NC, USA).

## 3. Results

### 3.1. Patients

**Figure 1:**
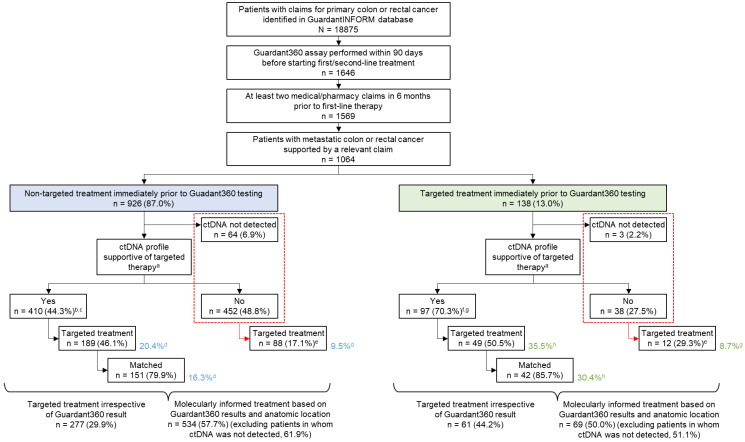
Disposition of patients. Percentages were calculated using the value in the preceding box as the denominator. ^a^ Presence of OncoKB level 1–2 or lack of R1; ^b^ 47.6% of samples with ctDNA present; ^c^ Excludes 48 patients with right-sided mCRC and *RAS* wild-type only; ^d^ Percentage calculated using non-targeted treatment (blue shaded box) as the denominator; ^e^ Percentage calculated using ‘No’ and ‘ctDNA not detected’ (outlined with red dashed box) as the denominator; ^f^ 71.9% of samples with ctDNA present; ^g^ Excludes 9 patients with right-sided mCRC and *RAS* wild-type only; ^h^ Percentage calculated using targeted treatment (green shaded box) as the denominator; Matched, patients who received a treatment matched to the molecular alterations revealed by the ctDNA assay; targeted, patients who received a molecularly targeted treatment.

A total of 18,875 patients with primary colon or rectal cancer were identified in the GuardantINFORM database. Of these, a Guardant360 test was performed within 90 days of starting second- or third-line therapy in 1646 patients, 1569 of whom had at least two medical or pharmacy claims in the 6 months prior to first-line therapy. A total of 1064 patients with metastatic disease that could be confirmed by a relevant claim were analyzed (Figure 1). Subsequent treatment information was available for all 1064 patients.

Of 1064 patients, 642 patients underwent ctDNA testing prior to second-line treatment (second-line cohort) and 422 patients underwent testing prior to third-line treatment (third-line cohort). Overall, 62 of 642 patients in the second-line cohort received first-line targeted therapy and 76 of 422 patients in the third-line cohort received second-line targeted therapy before ctDNA testing. Patients who received prior targeted therapies were assumed to be more likely to have tumors with relevant genomic biomarkers; therefore, these two independent groups were combined (n = 138, 13% of all patients in the cohort; green box in Figure 1) to increase confidence in subsequent analyses, and results were compared to those from patients who had not received immediate prior targeted therapy (n = 926; blue box in Figure 1).

The median age for patients included in this analysis was 59 years and 44.8% were female. The anatomic location of the primary tumor as described in the claims was left-sided for 44.5%, right-sided for 18.9%, mixed for 25.8%, and unspecified for 10.8%. The most common treatments received immediately prior to ctDNA testing were chemotherapy alone (365/1064, 34.3%) or combination regimens including VEGF-targeted treatment, but without other targeted agents (561/1064). Targeted therapy alone or in combination with other medicines was administered to 138 patients, most of whom received EGFR-targeted treatment (125/138, 90.6%) (Table 1).

### 3.2. Overview of Alterations Detected

ctDNA was detected in plasma from 997 of 1064 patients tested (93.7%). Clinically actionable molecular profiles were detected in 507 samples (47.7% of all patients tested; 50.9% of samples with detectable ctDNA). Actionable profiles were more frequently detected in patients who had previously received targeted therapy (70.3% (97/138) of all samples tested; 71.9% (97/135) of samples with detectable ctDNA) than in patients who had received non-targeted therapy (44.3% (410/926); 47.6% (410/862) of samples with detectable ctDNA) (*p* < 0.001 for comparisons of all samples and samples with ctDNA detected), suggesting the persistence of actionable profiles after treatment (Figure 1).

Considering only the samples with ctDNA present, mutations in *RAS* genes (*KRAS* or *NRAS*) were found in 45.6% (455/997). The frequency of *KRAS* mutations was lower (21.5% vs. 45.8%, *p* < 0.001) but the frequencies of *BRAF* V600E (12.6% vs. 6.5%, *p* = 0.019) and *ERBB2* amplification (5.9% vs. 2.3%, *p* = 0.043) were higher among patients who had previously received targeted therapy than in patients who had received non-targeted therapy (Table 2). There was a non-significant trend for more *NRAS* mutations after targeted treatment (8.9% vs. 4.6%, *p* = 0.063). *KRAS* G12C was detected in 41 samples (4.1%). The assay also detected rare alterations such as *MET* exon 14 skipping and fusions involving *FGFR3*, *RET*, *ALK*, and *ROS1* for which targeted therapies exist in other diseases but did not meet the definition for actionability in this analysis.

### 3.3. Evaluation of Molecularly Informed Therapy

Irrespective of ctDNA test results, targeted therapy was administered to 338 of 1064 patients (31.8%) in the subsequent treatment line immediately after ctDNA testing. However, patients with samples harboring clinically actionable molecular alterations according to ctDNA were more likely to receive targeted therapy than those who did not have actionable profiles (46.9% (238/507) vs. 18.0% (100/557; including patients with nondetectable ctDNA), *p* < 0.001). Targeted therapy for actionable profiles was administered to 189 of 926 patients (20.4%) without targeted treatment prior to ctDNA testing and to 49 of 138 patients (35.5%) with an immediate past history of targeted treatment (Figure 1). Among patients with samples containing clinically actionable molecular profiles, the rates of targeted treatment were similar between those who had received recent targeted therapy (49/97, 50.5%) and those who had not (189/410, 46.1%, *p* = 0.502).

### 3.4. Types of Treatments Administered after ctDNA Testing

The types of treatments administered after ctDNA testing are summarized in Table 3.

Non-targeted therapies (chemotherapy and/or VEGF targeting agents) were administered to approximately two-thirds of patients (726/1064, 68.2%) and were considered molecularly informed in 56.2% (408/726). Among patients whose treatment was not molecularly informed, 50 samples lacked ctDNA and 269 samples had molecular profiles associated with eligibility for targeted therapy: 27 with *BRAF* V600E, 6 with *ERBB2* amplification, 1 with MSI-high, 1 with MSI-high and concurrent *BRAF* V600E, and 234 without *KRAS* or *NRAS* mutations. Of these 234 patients without *KRAS* or *NRAS* mutations, 195 had no previous exposure to EGFR-targeting agents.

EGFR-targeted therapy was administered to 233 patients (21.9%), of whom 156 (67.0%) received treatment and were considered to be molecularly informed by ctDNA testing and primary tumor site (Table 3). Patients whose treatment was classified as not molecularly informed included 14 with no ctDNA detected and 23 with RAS mutations identified in ctDNA. Eighteen patients without RAS mutations had right-sided colon cancer. Other patients had actionable mutations in ctDNA, including 21 with *BRAF* V600E and 1 with MSI-high.

Immune checkpoint inhibitors either alone or in combination with other agents were administered to 78 patients (7.3%), 16 (20.5%) of whom had evidence of MSI-high status in ctDNA. Among the remaining 62 patients, 3 had undetectable ctDNA, and 20 had alternative genomic targets: 4 with *BRAF* V600E, 2 with *ERBB2* amplification, and 14 without *RAS* mutations including 11 without prior EGFR antibody treatment. Of the remaining 39 patients treated with immunotherapy, 18 had not received all possible standard of care treatment including fluoropyrimidine, oxaliplatin, irinotecan, and VEGF-targeted therapy. For 21 patients who had exhausted standard of care therapies, information on tumor mutation burden was not available.

Among 16 patients treated with BRAF-targeted agents and 21 treated with ERBB2-targeted agents, ctDNA was unable to detect the relevant alteration in 3 and 2 patients, respectively.

### 3.5. Patient Outcomes

**Figure 2:**
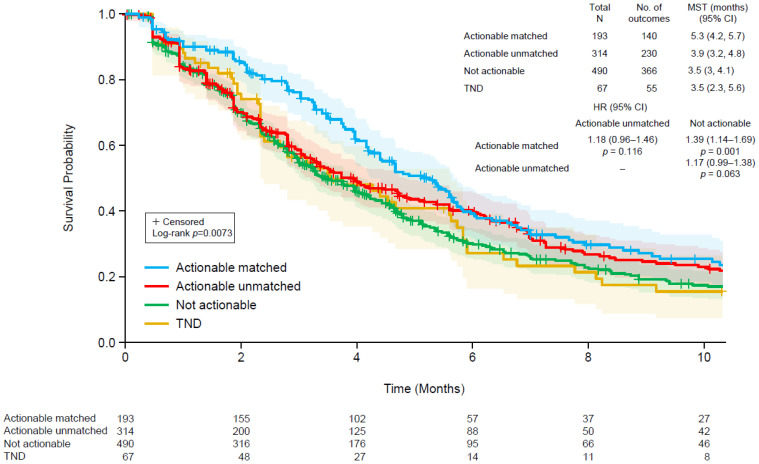
Kaplan–Meier plots of time to discontinuation of therapy following ctDNA testing using a Guardant360 assay in four groups: (1) patients with a clinically actionable genomic profile who received matched therapy (blue), (2) patients with a clinically actionable genomic profile who did not receive a matched therapy (red), (3) patients without a clinically actionable genomic profile (green), and (4) patients in whom ctDNA was not detected (brown). The shaded regions indicate 95% confidence intervals. CI, confidence interval; HR, hazard ratio; MST, median survival time.

The median TTD for patients treated in the second- and third-line settings after ctDNA testing were similar (4.2 vs. 3.8 months, *p* = 0.1764; Supplementary Figure S1). Therefore, we included patients from both groups in the subsequent analyses to increase the sample size and to allow for a more robust analysis. As shown in Figure 2, we assessed TTD in the following four groups: (1) patients with a clinically actionable molecular profile according to ctDNA who received matched therapy, (2) patients with a clinically actionable molecular profile who did not receive matched therapy, (3) patients without a clinically actionable genomic profile, and (4) ctDNA not detected. TTD was significantly different among the four groups (log-rank *p* = 0.0073). The median TTD was longest among patients with an actionable profile who received matched therapy (5.3 months) and similar for the other groups (3.9 months for actionable profiles without matched therapy and 3.5 months each for non-actionable profiles or those without ctDNA detected). Among patients with actionable ctDNA profiles, there was a non-significant trend for longer TTD for those who received matched versus unmatched therapy (hazard ratio 1.18, 95% confidence interval, 0.96–1.46; *p* = 0.116). An analysis of overall survival, separated by line of therapy due to clear differences in outcomes favoring longer duration in the earlier line of treatment, revealed no clear patterns among the four groups (Supplementary Figure S2).

**Figure 3:**
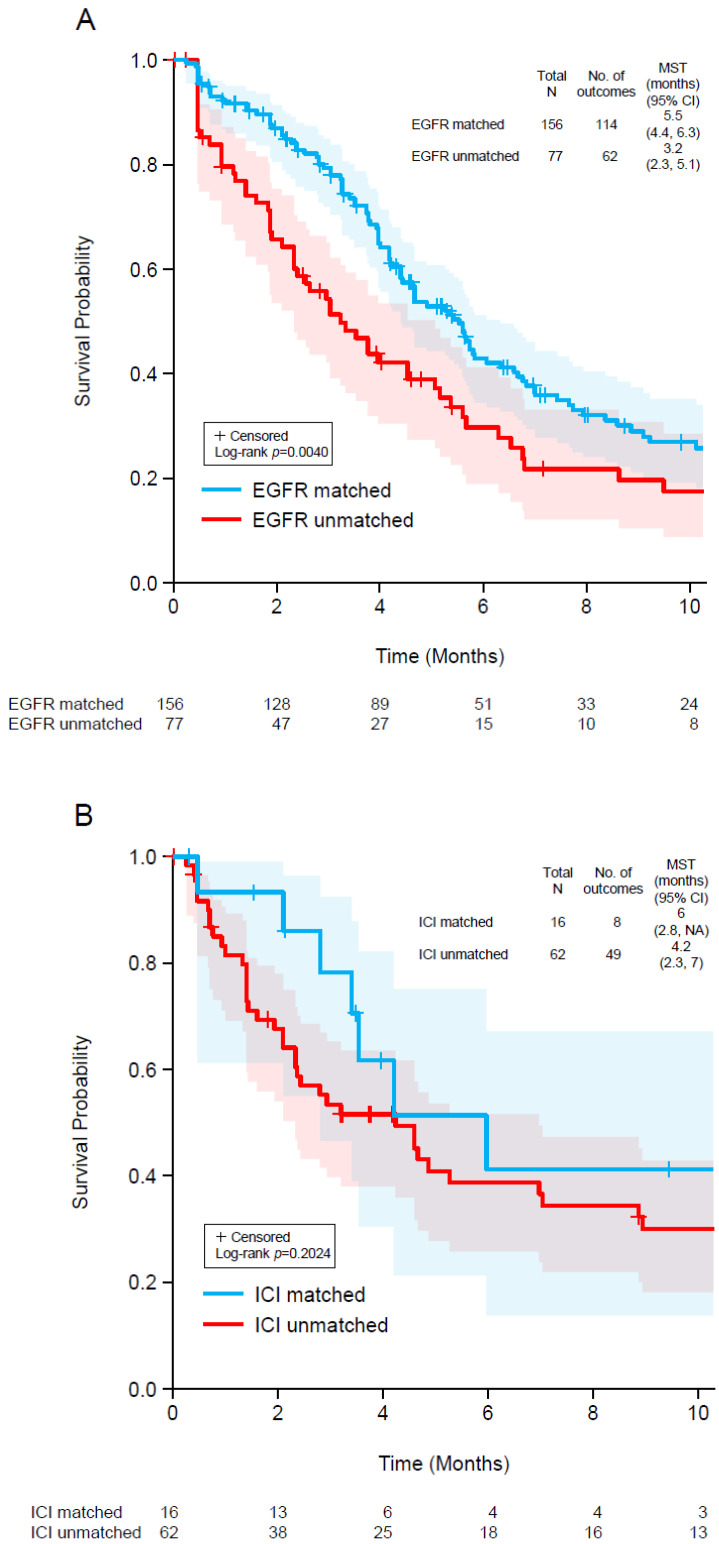
Kaplan–Meier plots of time to discontinuation of EGFR-targeted therapy (**A**) and immune checkpoint inhibitor therapy (**B**) in two groups: (1) patients with a clinically actionable genomic profile who received matched targeted therapy (blue) and (2) patients whose genomic profile did not support the therapy used. The shaded regions indicate 95% confidence intervals. CI, confidence interval; EGFR, epidermal growth factor receptor; ICI, immune checkpoint inhibitor; MST, median survival time.

We also analyzed TTD for the 233 patients who received EGFR-targeted therapy (Figure 3A) and the 78 patients treated with immune checkpoint inhibitors (Figure 3B) because these were the most common types of targeted therapy used after ctDNA testing. We classified patients as those whose ctDNA profiles supported the choice of the targeted treatment (matched) and those whose profiles did not support such treatment (unmatched). Among patients who received EGFR-targeted therapy, the median TTD was significantly longer in those with matched treatment (5.5 months) than in those with unmatched treatment (3.2 months; log-rank *p* = 0.0040; Figure 3A). Among patients who received immunotherapy, the median TTD tended to be longer in those with matched treatment (6.0 months) than in those with unmatched treatment (4.2 months; log-rank *p* = 0.2024; Figure 3B).

## 4. Discussion

For patients with mCRC and disease progression, we analyzed a real-world medical claims database combined with ctDNA test results to assess the types of therapeutic decisions made based on ctDNA profiling and the clinical outcomes associated with those decisions.

The assay used in this analysis detected ctDNA in 93.7% of the samples, a similar result to that previously reported for plasma samples from a large cohort of mCRC patients tested in routine clinical practice [34]. Clinically actionable ctDNA profiles were detected in approximately half (507/1064, 47.7%) of all patients tested, not only among those who had received targeted therapy immediately prior to testing (97/138, 70.3%) but also in those with disease progression after non-targeted therapy (410/926, 44.3%).

The frequencies of informative genomic alterations detected in this study were generally consistent with those described in previous studies [35,36,37,38,39]. *KRAS* mutations were the most common (424 of 997 samples with ctDNA detected, 42.5%), followed by *BRAF* V600E (7.3%), *NRAS* (5.2%), and *ERBB2* amplification (2.8%). MSI-high assessment was added to the ctDNA assay in 2018, but the database includes patients tested from 2014; therefore, the observed proportion of samples with MSI-high status (2.0%) most likely underestimates the true prevalence, which is expected to be up to 5% [40]. Among patients with detectable ctDNA, the proportion of patients with *KRAS* mutations was lower in those who had previously received targeted therapy (21.5%) than in those who had not (45.8%), presumably due to prior selection for *RAS* wild-type profiles in consideration of EGFR-targeted regimens, which were the most commonly used targeted therapies prior to ctDNA testing. However, there was a trend for more *NRAS* mutations among patients exposed to prior targeted treatment (8.9%) vs. patients not exposed to prior targeted therapy (4.6%) (*p* = 0.063). As expected, prior targeted therapy, which was overwhelmingly EGFR-targeted, increased the proportion of cancers with *BRAF* V600E (12.6% vs. 6.5%, *p* = 0.019) and *ERBB2* amplification (5.9% vs. 2.3%, *p* = 0.043).

Targeted therapies were more likely to be administered to patients with actionable ctDNA profiles (238/507, 46.9%) than to those without actionable profiles (100/557, 18.0%), but only slightly more than one-third of patients with actionable profiles received appropriately matched therapy (38.1%, 193/507). Excluding patients in whom ctDNA could not be detected, molecularly informed therapy, targeted or not, was administered to 60.5% of patients (603/997). However, because the claims database used in this study does not include results of other biomarker tests, we cannot exclude the possibility that some treatment decisions were molecularly informed by other assays.

In one recent study of 78 patients with mCRC, alterations that could be targeted by available therapies were detected using ctDNA in 59 (75.6%), and 45 satisfied OncoKB criteria in a manner similar to that employed in our study [36]. In another study of 94 patients who underwent ctDNA testing, non-synonymous mutations were detected in 74 patients (79%), of whom 69 (73%) had potentially actionable alterations [37]. In a study that used cell-free DNA (cfDNA) samples from 128 patients with mCRC, somatic alterations were detected in 100 (78%), of which 50% had potentially actionable alterations [41]. However, even under controlled study circumstances, targeted therapy may not be administered to all potentially eligible patients. For example, in the MOSCATO 01 trial in which NGS was used to guide treatment decisions in a cohort of 948 patients with advanced cancers who underwent tumor biopsy, a molecular portrait was developed for 843. An actionable alteration was found in 411 patients, but only 199 (48.4%) received a targeted therapy matched to the alteration [42].

Our findings support the clinical utility of matched therapy for mCRC patients with actionable molecular profiles detected by ctDNA. Median TTD, a surrogate for progression-free survival, was longest for patients with actionable profiles who received matched targeted treatment (5.3 months) than in those with actionable profiles who received unmatched treatment (3.9 months). Patients without actionable ctDNA profiles or those in whom ctDNA was not detected had the shortest median TTD (3.5 months each). These results are consistent with those of other studies demonstrating the importance of matching treatment to tissue-based molecular profiles [36,37,42]. The survival benefit of molecularly matched therapy based on tissue-based testing was previously reported [43,44,45,46]. However, the utility of ctDNA genotyping for molecularly matched therapy in real-world clinical practice, especially for mCRC patients following prior targeted therapies, has not been fully addressed. The clinical outcomes of 35 mCRC patients from a single institution suggested that matched therapy based on ctDNA results achieved a higher tumor response rate than unmatched therapy, with a trend for longer progression-free survival [37]. Our study, which included patients from academic and community-based settings, confirmed the usefulness of ctDNA genotyping for identification of patients who would benefit from targeted therapies.

One important role of ctDNA testing prior to later-line therapies is identification of resistance mutations. For example, testing identified patients who are less likely to respond to EGFR-targeted therapy. We observed that TTD following EGFR-targeted therapy was significantly prolonged when treatment was matched to the ctDNA profile. Furthermore, even though the absence of detectable ctDNA in plasma samples of mCRC patients is associated with a better prognosis [47], the TTD was longer in patients with a clinically actionable genomic profile who received matched therapy than in ctDNA-negative patients. This underscores the efficacy of matched targeted therapy based on ctDNA genotyping.

Although ctDNA testing identified patients who are less likely to benefit from EGFR-targeted treatments, this class of therapy appeared to be under-utilized in this cohort. A small proportion of patients received EGFR-targeted therapies immediately prior to ctDNA testing (125/1064, 11.7%), although it is expected that about 40% should be eligible [9,12]. After ctDNA testing, 248 patients with molecular and clinical profiles supporting EGFR-targeted therapies received other types of therapies instead. For most of these, there was no prior history of EGFR-targeted treatment. Although chemotherapy with or without anti-VEGF therapy can be effective in this setting, clinicians and patients should be aware of alternative treatment options. It is also important to consider that ctDNA can identify patients who should not receive EGFR-targeted therapy. The median TTD was shorter for patients treated with EGFR-targeted agents with unfavorable ctDNA results (77/233 treated; 3.2 months) compared with patients whose treatment was matched to the ctDNA profile (5.5 months).

Most patients treated with immune checkpoint inhibitors did not have MSI-high detected by ctDNA (62/78, 79.5%). There was a trend toward longer median TTD among patients treated with immunotherapy and MSI-high samples (6 months) compared to those without MSI-high (4.2 months). However, this was not statistically significant due to the small number of patients with MSI-high and treated with immune checkpoint inhibitors. The ctDNA assay used in this study introduced MSI-high assessment several years into the observation period and did not include all potential genomic information that could inform the suitability of immunotherapy. For example, *POLD1* and *POLE* are not in the panel. Mutations in these genes, which occur in <1% of cases, are associated with response to immune checkpoint inhibitors [40,48]. Furthermore, the assay used in this study did not assess for the presence of high tumor mutation burden, which can be detected in approximately 3% of patients with mCRC without MSI-high [40]. The US FDA approved pembrolizumab for the treatment of solid tumors with a high tumor mutation burden (defined as ≥10 mutations/megabase), as determined by an FDA-approved test, in patients with disease progression after prior treatment and who have no satisfactory alternative treatment options [49]. However, standard treatment was not exhausted by over one-quarter of patients without MSI-high (18/62) who received an immune checkpoint inhibitor. Furthermore, even among mCRC patients with a high tumor mutation burden in the absence of MSI-high, the effectiveness of immunotherapy remains controversial [39,50].

There are some limitations of this study. In particular, the database lacked complete clinical information, such as performance status of the patients, physician-assessed tumor response, and results of concurrent biomarker testing that may have informed clinical decisions. Furthermore, because all treatments were identified using healthcare claims data, the database did not evaluate participation in clinical trials. Despite these factors, the genomic landscape observed in this study is generally consistent with that reported in other studies [51,52].

This study was designed to determine the proportion of patients for whom the potential use of targeted therapy would be appropriate following ctDNA testing. Therefore, our definition of molecularly informed therapy was intentionally narrow. Many of the treatment decisions made in this real-world setting may be valid, considering additional clinical information that was not available when these patients were treated. Tumor molecular information is necessary to make informed treatment decisions for patients with mCRC, but it should not be the only consideration.

In summary, this analysis of a real-world database demonstrates the clinical value of plasma-based comprehensive genomic profiling in mCRC patients with disease progression after one or two lines of prior therapy. Analysis of ctDNA identifies patients who are candidates for new or further targeted therapy, regardless of prior treatment history. Most importantly, in standard clinical practice, patients who received targeted treatments that matched their ctDNA test results had more favorable treatment outcomes than patients who received unmatched therapies. The modest improvement in TTD with the use of matched therapy for actionable alterations underscores the need for continued improvement in the identification of predictive biomarkers and the development of therapeutic agents to target such biomarkers. Future analyses of real-world clinical practice databases will help to further characterize the evolving role of ctDNA testing and its potential to influence the clinical outcomes of people living with advanced CRC.

## Figures and Tables

**Table 1:** Treatments immediately prior to ctDNA testing.

Class of Treatment	Second-Line Cohort (n = 642)		Third-Line Cohort (n = 422)		Total (n = 1064)	
	n	%	n	%	n	%
Any non-targeted *	580	90.3%	346	82.0%	926	87.0%
Chemotherapy only	262	40.8%	103	24.4%	365	34.3%
VEGF inhibitor (with or without chemotherapy, no targeted treatment)	318	49.5%	243	57.6%	561	52.7%
Any targeted	62	9.7%	76	18.0%	138	13.0%
EGFR-targeted	56	8.7%	69	16.4%	125	11.7%
BRAF-targeted	0	0.0%	3	0.7%	3	0.3%
ERBB2-targeted	3	0.5%	1	0.2%	4	0.4%
Other targeted	1	0.2%	0	0.0%	1	0.1%
Immune checkpoint inhibitor	3	0.5%	5	1.2%	8	0.8%

* Chemotherapy alone or VEGF inhibitor with or without chemotherapy. VEGF, vascular endothelial growth factor; EGFR, epidermal growth factor receptor; BRAF, B-Raf proto-oncogene serine/threonine-protein kinase; ERBB2, erb-b2 receptor tyrosine kinase 2.

**Table 2:** Frequencies of clinically informative alterations detected by ctDNA.

Mutation	Prior Therapy: Targeted			Prior Therapy: Non-Targeted			*p*-Value *		Total		
	n	All Tested (%)	With ctDNA (%)	n	All Tested (%)	With ctDNA (%)	All Tested	With ctDNA	n	All Tested (%)	With ctDNA (%)
n		138	135		926	862				1064	997
*KRAS* mutation ^†^	29 ^‡^	21.0%	21.5%	395 ^§^	42.7%	45.8%	<0.001	<0.001	424	39.8%	42.5%
*NRAS* mutation ^†^	12	8.7%	8.9%	40	4.3%	4.6%	0.044	0.063	52	4.9%	5.2%
Any *RAS* mutation ^†^	33	23.9%	24.4%	422 ^‡^	45.6%	49.0%	<0.001	<0.001	455	42.8%	45.6%
*BRAF* V600E ^†^	17	12.3%	12.6%	56	6.0%	6.5%	0.011	0.019	73	6.9%	7.3%
*ERBB2* amplification ^†^	8	5.8%	5.9%	20	2.2%	2.3%	0.021	0.043	28	2.6%	2.8%
*MSI-H ^†^*	3	2.2%	2.2%	17	1.8%	2.0%	0.736	0.745	20	1.9%	2.0%
*NTRK1* fusion ^†^	1	0.7%	0.7%	0	0	0	0	0	1	0.1%	0.1%
*RET* fusion	1	0.7%	0.7%	1	0.1%	0.1%	0	0	2	0.2%	0.2%
*ALK* fusion	1	0.7%	0.7%	0		0	0	0	1	0.1%	0.1%
*ROS1* fusion	0	0	0	1	0.1%	0.1%	0	0	1	0.1%	0.1%
*FGFR3* fusion	1	0.7%	0.7%	1	0.1%	0.1%	0	0	2	0.2%	0.2%
*MET* exon 14 skipping	0	0	0	1	0.1%	0.1%	0	0	1	0.1%	0.1%

Some samples had multiple alterations. * Targeted vs. non-targeted, ^†^ OncoKB level 1–2 or R1, ^‡^ Includes 5 patients with *KRAS* G12C (17.2% of *KRAS* mutations); ^§^ Includes 36 patients with *KRAS* G12C (9.1% of *KRAS* mutations).

**Table 3:** Systemic treatments after ctDNA testing.

	Second-Line Cohort (n = 642)		Third-Line Cohort (n = 422)		Total (n = 1064)	
Class of Treatment	n	Informed, n (%)	n	Informed, n (%)	n	Informed, n (%)
Chemotherapy only	184	96 (52.2%)	105	68 (64.8%)	289	164 (56.7%)
VEGF inhibitor (with or without chemotherapy, no targeted treatment)	275	148 (53.8%)	162	96 (59.3%)	437	244 (55.8%)
Any non-targeted *	459	244 (53.2%)	267	164 (61.4%)	726	408 (56.2%)
EGFR-targeted	132	77 (58.3%)	101	79 (78.2%)	233	156 (67.0%)
BRAF-targeted	9	8 (88.9%)	7	5 (71.4%)	16	13 (81.3%)
ERBB2-targeted	11	11 (100%)	10	8 (80.0%)	21	19 (90.5%)
Other targeted	1	1 (100%)	0	0	1	1 (100%)
Immune checkpoint inhibitor	37	11 (32.4%)	41 ^†^	5 (12.2%)	78	16 (20.5%)
Any treatment	642	346 (53.9%)	422	257 (60.9%)	1064	603 (56.7%)

Molecularly informed therapy was defined as targeted therapy for an actionable molecular profile or as non-targeted therapy in the absence of an actionable profile. If ctDNA were not detected, the cases were classified as not molecularly informed for any treatment. Other than those treated with chemotherapy only, some patients may have received multiple therapies.* Chemotherapy alone or VEGF inhibitor with or without chemotherapy, ^†^ 21 patients without an actionable profile received an immune checkpoint inhibitor after 5-FU, irinotecan, and oxaliplatin VEGF, vascular endothelial growth factor; EGFR, epidermal growth factor receptor; BRAF, B-Raf proto-oncogene serine/threonine-protein kinase; ERBB2, erb-b2 receptor tyrosine kinase 2.

## Data Availability

The datasets generated during and/or analyzed during the current study are not publicly available and cannot be shared due to the use of a third-party healthcare claims database. Researchers interested in replicating our study or pursuing new research topics should contact Guardant Health (https://guardanthealth.com/products/biopharma-solutions/real-world-evidence/, accessed on 18 March 2022) directly.

## Supplementary Materials

The following are available online at https://www.mdpi.com/article/10.3390/curroncol29050277/s1, Figure S1: Kaplan–Meier plots of time to treatment discontinuation following second-line (blue) or third-line (red) therapy after Guardant360 testing. The shaded regions indicate 95% confidence intervals. CI, confidence interval; MST, median survival time, Figure S2: Kaplan–Meier plots of overall survival following second- (A) or third-(B) line therapy in patients who underwent Guardant360 testing: (1) patients with a clinically actionable genomic profile who received matched therapy (blue); (2) patients with a clinically actionable genomic profile who did not receive matched therapy (red); (3) patients without a clinically actionable genomic profile (green); and (4) patients in whom ctDNA was not detected (brown). The shaded regions indicate 95% confidence intervals.
