# Supplementary material for: Comprehensive Genomic Profiling of Circulating Tumor DNA in Patients with Previously Treated Metastatic Colorectal Cancer: Analysis of a Real-World Healthcare Claims Database
Source: Curr Oncol. 2022 May 9;29(5):3433–48. doi: 10.3390/curroncol29050277 (PMC9139639; doi:10.3390/curroncol29050277)
Supplement: Supplementary file 1 [file curroncol-29-00277-s001.zip › curroncol-1665546-supplementary.pdf]

Current Oncology

MDPI

Supplementary materials

Comprehensive Genomic Profiling of Circulating Tumor DNA in Patients with Previously Treated Metastatic Colorectal Cancer: Analysis of a Real-World Healthcare Claims Database

Yoshiaki Nakamura, Steven Olsen, Nicole Zhang, Jiemin Liao and Takayuki Yoshino

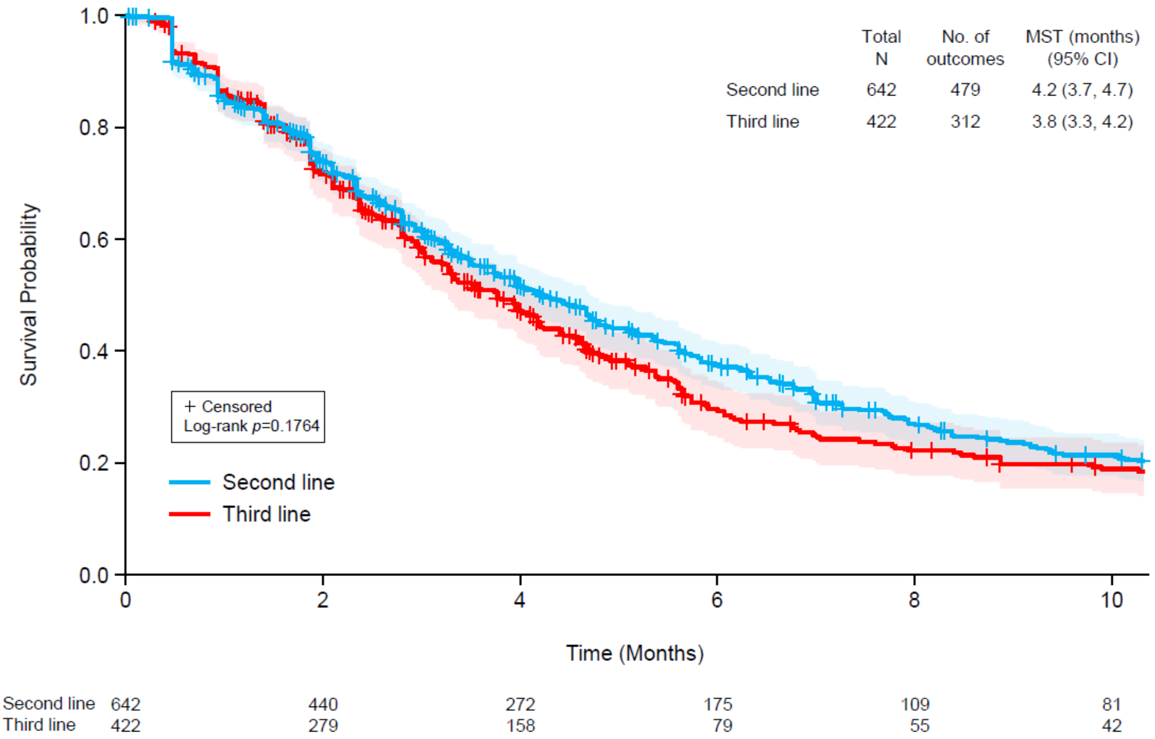

Figure S1. Kaplan–Meier plots of time to treatment discontinuation following second-line (blue) or third-line (red) therapy after Guardant360 testing. The shaded regions indicate 95% confidence intervals. CI, confidence interval; MST, median survival time.

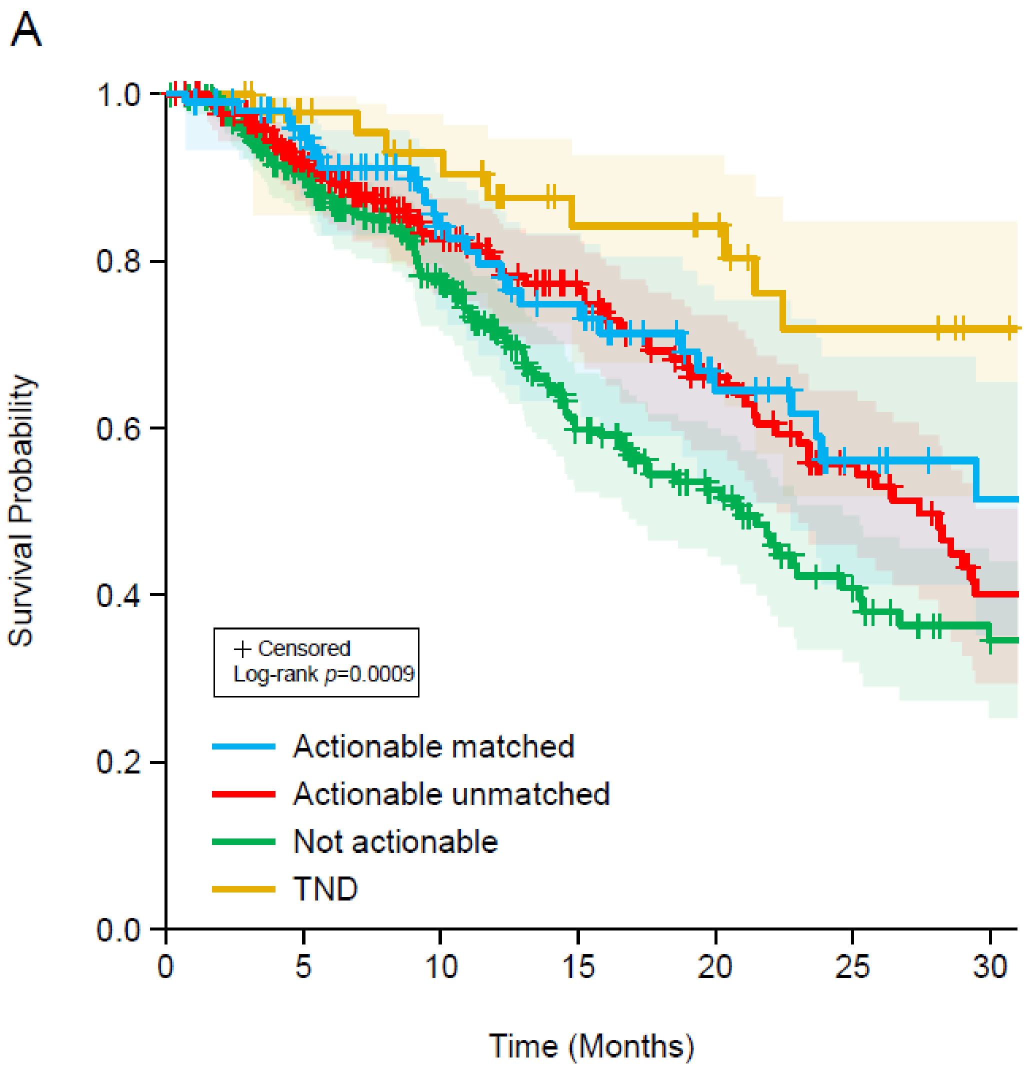

Actionable matched	101	85	57	44	27	16	11
Actionable unmatched	213	167	119	91	61	39	24
Not actionable	280	215	145	86	54	30	18
TND	48	41	36	25	23	17	14

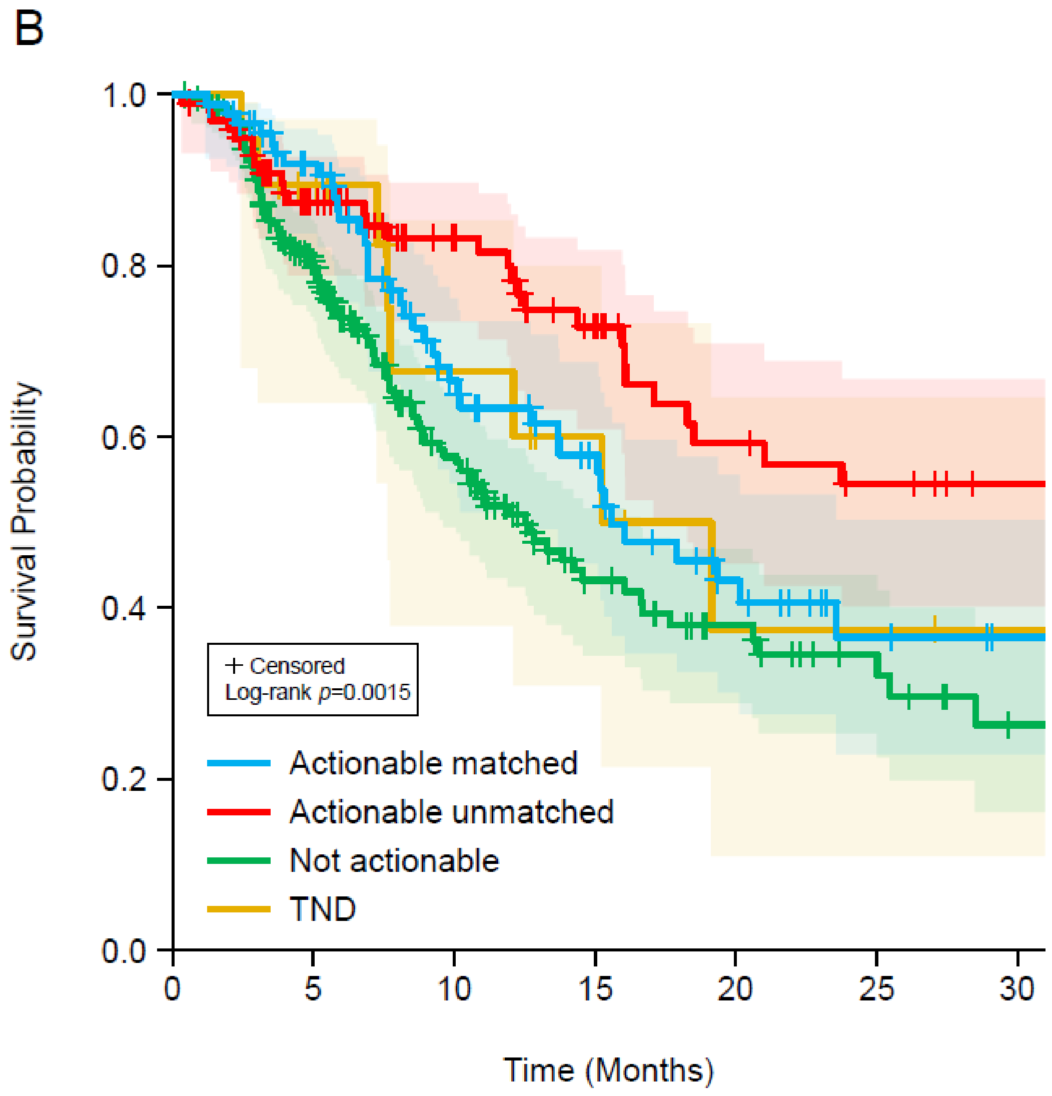

Actionable matched	92	73	42	29	17	9	6
Actionable unmatched	101	71	52	36	26	22	18
Not actionable	210	138	73	34	23	14	7
TND	19	15	9	6	3	3	2

Figure S2. Kaplan–Meier plots of overall survival following second- (A) or third-(B) line therapy in patients who underwent Guardant360 testing: (1) patients with a clinically actionable genomic profile who received matched therapy (blue); (2) patients with a clinically actionable genomic profile who did not receive matched therapy (red); (3) patients without a clinically actionable genomic profile (green); and (4) patients in whom ctDNA was not detected (brown). The shaded regions indicate 95% confidence intervals.
